# Perinatal insults and neurodevelopmental disorders may impact Huntington's disease age of diagnosis

**DOI:** 10.1016/j.parkreldis.2018.05.016

**Published:** 2018-10

**Authors:** Melinda Barkhuizen, Filipe B. Rodrigues, David G. Anderson, Bjorn Winkens, REGISTRY Investigators of the European Huntington's Disease Network, Edward J. Wild, Boris W. Kramer, A.W.Danilo Gavilanes

**Affiliations:** a Department of Pediatrics, Maastricht University Medical Center (MUMC), Maastricht, The Netherlands; b Department of Translational Neuroscience, School of Mental Health and Neuroscience (MHeNs), Maastricht University, Maastricht, The Netherlands; c DST/NWU Preclinical Drug Development Platform, North-West University, Potchefstroom, South Africa; d Huntington's Disease Centre, Institute of Neurology, University College London, London, UK; e Clinical Pharmacology Unit, Instituto de Medicina Molecular, Lisbon, Portugal; f Laboratory of Clinical Pharmacology and Therapeutics, Faculty of Medicine, University of Lisbon, Lisbon, Portugal; g Department of Neurology, University of the Witwatersrand Donald Gordon Medical Centre, Johannesburg, South Africa; h Department of Methodology and Statistics, Care and Public Health Research Institute (CAPHRI), Maastricht University, Maastricht, The Netherlands; i School of Oncology and Developmental Biology, Maastricht University, Maastricht, The Netherlands; j Institute of Biomedicine, Facultad de Ciencias Médicas, Universidad Católica de Santiago de Guayaquil, Ecuador

**Keywords:** Huntington's disease, Neonatal, Developmental disorders, Epidemiological, Modifier

## Abstract

**Introduction:**

The age of diagnosis of Huntington's disease (HD) varies among individuals with the same *HTT* CAG-repeat expansion size. We investigated whether early-life events, like perinatal insults or neurodevelopmental disorders, influence the diagnosis age.

**Methods:**

We used data from 13,856 participants from REGISTRY and Enroll-HD, two large international multicenter observational studies. Disease-free survival analyses of mutation carriers with an *HTT* CAG repeat expansion size above and including 36 were computed through Kaplan-Meier estimates of median time until an HD diagnosis. Comparisons between groups were computed using a Cox proportional hazard survival model adjusted for CAG-repeat expansion length. We also assessed whether the group effect depended on gender and the affected parent.

**Results:**

Insults in the perinatal period were associated with an earlier median age of diagnosis of 45.00 years (95%CI: 42.07–47.92) compared to 51.00 years (95%CI: 50.68–51.31) in the reference group, with a CAG-adjusted hazard ratio of 1.61 (95%CI: 1.26–2.06). Neurodevelopmental disorders were also associated with an earlier median age of diagnosis than the reference group of 47.00 years (95% CI: 43.38–50.62) with a CAG-adjusted hazard ratio of 1.42 (95%CI: 1.16–1.75). These associations did not change significantly with gender or affected parent.

**Conclusions:**

These results, derived from large observational datasets, show that perinatal insults and neurodevelopmental disorders are associated with earlier ages of diagnosis of magnitudes similar to the effects of known genetic modifiers of HD. Given their clear temporal separation, these early events may be causative of earlier HD onset, but further research is needed to prove causation.

## Highlights

• The CAG-repeat expansion does not fully explain the onset of Huntington's disease (HD).
• In two large observational studies, birth complications were associated with the onset of HD.
• Persons with disorders of brain development also had an earlier diagnosis of HD.
• Early-life factors may be an environmental modifier of HD.

## 1 Introduction

Huntington's disease (HD) is a progressive neurodegenerative disease characterized by motor, cognitive and behavioral symptoms. In Europe, North America, and Australia the overall prevalence is 5.70 cases per 100,000 [1] and is expected to increase by approximately 15–20% per decade [2]. There is no cure and little evidence to support symptomatic treatment [3].

The length of the CAG-repeat expansion in the *HTT* gene (Hugo Gene Nomenclature ID: 4851) is the largest determinant of the age of diagnosis of disease, accounting for approximately 67% of the overall variation [4]. However, there is still substantial variability in the age of diagnosis after controlling for repeat expansion length, which by definition is due to some combination of other genetic or environmental factors or the interaction between genetic and environmental factors. Variation in certain loci has been shown to hasten the onset of disease by up to 6 years or delay it by 1.4–1.6 years [5]. In a large Venezuelan kindred, as much as 41% of the age of diagnosis variability in HD families was attributed to environmental factors not shared by family members [6]. Identifying environmental modifiers which may be targeted to delay the age of onset in pre-manifest gene expansion carriers (GEC) is critical to reducing the burden of HD.

The inheritance of an HD mutation initiates a lifelong pathogenic process, which is eventually followed by symptom onset and clinical diagnosis [4]. There is growing evidence that key pathological processes in HD have their origin early in life [7]. The huntingtin protein (HTT) is important for neurogenesis and neuronal migration [8], and a complete loss of HTT function during embryonic development is lethal [7]. Disruption of HTT function *in utero*, through reduced expression of normal HTT, or expressing mutant poly-CAG HTT, alters the morphology of cortical neurons in adulthood and can cause cortical and striatal degeneration during aging [9,10].

The aim of this study was to determine whether early-life events alter the natural history of HD. We investigated the association of perinatal insults and neurodevelopmental disorders with age of diagnosis of HD in two large multicenter international longitudinal cohorts [11,12] using survival analysis methodology. Perinatal insults have been linked to deficits in functional domains affected in HD, such as cognition, locomotion, behavior or sensory development in approximately 40% of survivors in the general population [13]. We further examined whether associations depend on the gender of the participants or the gender of the affected parent, since it is possible that these may influence any relationship with early life events and age of diagnosis in HD. The gender of the affected parent may have an effect on age of diagnosis in HD in two ways: the CAG-repeat expansion is more likely to further expand during transmission from an affected father, which decreases the age of onset in his offspring [14]. Alternately, it could also be hypothesized that GEC whose mothers were also carriers of the abnormal gene might have experienced effects during embryonic and fetal development, which were not experienced by GEC that inherited the mutated gene from their fathers; and this might exert an independent effect on HD disease-free survival, either via the perinatal and neurodevelopmental factors we examined here, or through other routes. We hypothesized that a perinatal insult and/or a neurodevelopmental disorder, and an HD positive genotype could have additive damaging effects, which could manifest as an earlier disease diagnosis.

## 2 Materials and methods

We followed STROBE guidelines for reporting epidemiological results and SAMPL guidelines for reporting statistical findings according to the suggested guidelines of the EQUATOR Network.

### 2.1 Ethical approval and reporting guidelines

This study and its contributing works were performed in accordance with the declaration of Helsinki and approved by the local ethics committees for each study site contributing to REGISTRY (NCT01590589) and Enroll-HD (NCT01574053). All participants gave informed written consent. Participants lacking consenting capacity had consent given on their behalf as requested by country-specific ethical standards. Only data from persons above and including 21 years of age were included.

### 2.2 Datasets, study designs, and participants

All participants were part of the European Huntington's Disease Network's (EHDN) multicenter, European, prospective observational study – REGISTRY (V2 and V3) [11]; or part of the Enroll-HD (2016 release) [12]. 14,893 participants from 165 study sites in 21 European countries were enrolled in REGISTRY between 2004 and 2016. Enroll-HD succeeded REGISTRY and also included participants from North America, Latin America, and Australasia enrolled between 2012 and 2016. The Enroll-HD 2016 release contained the data of 8714 participants, including 3598 participants previously enrolled in REGISTRY. These longitudinal cohorts include manifest and pre-manifest GEC, as well as healthy controls and individuals at risk of HD. For our analysis, we only included GEC with: a CAG-repeat expansion length above and including 36 repeats on the major allele; an age at diagnosis or an age at last visit above and including 21 years; and available co-morbidities data. We removed duplicate records from the Enroll-HD participants who were also enrolled in REGISTRY. This limited the number of participants to 7686 manifest GEC and 2069 pre-manifest GEC from REGISTRY, and 2892 manifest and 1209 pre-manifest GEC from Enroll-HD. The age of diagnosis of HD variable was present in both databases, and carried over for the participants in REGISTRY, which were also included in Enroll-HD, and can thus be presumed to be recorded with comparable criteria in both studies. Since the variables of interest were similar between studies, as were the study designs, the two datasets were combined into one large dataset after excluding duplicate records. The final dataset for calculating the influence of age at diagnosis included the data of 10,578 manifest and 3278 pre-manifest GECs. For a secondary analysis on the influence of gender of the affected parent on the model, data were available for 7271 manifest and 3207 pre-manifest GECs. The amounts of participants included in each stage are shown in the Supplementary Fig. 1.

### 2.3 Identification of perinatal complications and neurodevelopmental disorders

Two perinatal investigators (M.B., A.G.) examined all the comorbidities listed in each database to identify perinatal insults and neurodevelopmental disorders. Perinatal insults (MeSH ID: D054238) were defined as insults which likely occurred between 28 weeks of gestation to 28 days after birth. Where recorded, the age at the event and ICD10 codes were used to describe events that occurred during the neonatal period, as opposed to complications of pregnancy described in the records of the mother. Dates of the adverse events were not recorded for all participants and where recorded, it was sometimes possible to narrow the insult down to the first year of life but not the first 28 days after birth. Conditions of this kind that were additionally listed as ‘intrauterine’, ‘perinatal’ or ‘neonatal’ were considered as having occurred within the perinatal time-frame.

For neurodevelopmental disorders, the co-morbidities records were screened for neurodevelopmental disorders included in the DSM-5 and ICD10 classification. This list includes conditions such as neurocognitive disorders, communication and language deficits, autism, and attention deficit hyperactivity disorder. The age-limit for neurodevelopmental disorders was set at 20 years and attention deficits above this age and psychiatric conditions, such as schizophrenia were excluded. We further looked at medication-use records for methylphenidate (and brand names) prescribed for ADHD or attention disturbances (and not for apathy, irritability, somnolence or psychiatric disturbances, etc.) prior to 20 years of age. We excluded tics and Tourette's syndrome as these may mimic the symptoms of HD. The list was supplemented with conditions listed in reviews on the neurodevelopmental outcomes of perinatal insults [13,15]. Non-specific disorders, which could also have an adulthood onset, such as seizures, psychiatric complaints, hearing loss and visual loss, with the exception of the pediatric visual disorder strabismus, were excluded to limit the statistical noise.

### 2.4 Statistical analysis

To determine participants' age of diagnosis, we used the Kaplan-Meier product limit method. A single estimate of the age of diagnosis in years (median and 95% confidence interval [95% CI]) was derived for each group of participants for each of the databases. For pre-manifest GECs, we used the latest visit date in their profile as an age of diagnosis-free survival. The primary analysis was done with the merged and de-duplicated dataset, and secondary sensitivity analyses were done with REGISTRY and Enroll-HD datasets independently.

The associations of perinatal insults and neurodevelopmental disorders with HD age of diagnosis were adjusted for CAG-repeat expansion length on the major allele through a Cox proportional hazards model. The assumption for the proportionality of hazard ratios (HR) was tested with Schoenfeld residuals and with time-dependent covariates. The overall proportional hazard assumption was not violated for analyzing time until diagnosis by the group, as the time-dependent covariates (p = 0.385), as well as Schoenfeld residuals, were not significant (all p = 0.188). Participants were grouped into three groups: perinatal insults, neurodevelopmental disorders, and the reference group consisting of the remaining participants (Table 1, Table 2). HR and 95%CI were generated for each of the groups, compared to the reference group. As a sensitivity measure, we recalculated the HRs in the individual absence of the most common perinatal insults and neurodevelopmental disorders. Additionally, we investigated whether the association seen per group depended on the gender of the participant or the gender of the parent from whom the mutation was inherited. The merged cohort was used for this analysis. The participant gender info was available for all participants; however, affected parent data was only available for a subset (Supplementary Fig. 1). Therefore, we first performed a multiple imputation (MI) method for the missing values of an affected parent using all the variables included in the Cox regression model as a predictor. After the MI, where the maximum number of iterations was set to 20, 30 complete datasets were created and Cox regression analysis was applied to each dataset and then pooled. To assess association modification we calculated interaction terms between group and gender, and between the group and affected parent in the model of the CAG adjusted HR. If the interaction term with gender and/or affected parent was statistically significant, we expressed the group effect for each level of gender and/or affected parent with the corresponding CAG-adjusted HR and 95%CI. As a sensitivity analysis, we also performed the complete case analysis, by applying the same model to only participants with complete information. We compared the pooled results after MI to the results from the complete case analysis.

**Table 1:** Description of the comorbidities included from the REGISTRY and Enroll-HD cohorts, divided into perinatal insults and neurodevelopmental disorders. N, the number of comorbidities; %, the percentage of comorbidities.

	Registry % (N)	Enroll-HD % (N)	Combined percentage % (N)
**Perinatal insults**			
Birth injury, birth asphyxia, apnea or meconium aspiration	33.78% (25)	17.65% (3)	30.76% (28)
Preterm birth	22.97% (17)	0% (0)	18.68% (17)
Kernicterus	13.51% (10)	5.88% (1)	12.09% (11)
Meningitis, encephalitis	8.11% (6)	17.65% (3)	9.89% (9)
Perinatal hematological disorder, Rh isoimmunization of fetus, transient neutropenia	6.76% (5)	5.88% (1)	6.59% (6)
Others (Acquired periventricular cysts, personal history of conditions arising in perinatal period, atelectasis of newborn, convulsions, intestinal perforation, hyperthermia, gestational diabetes of mother)	5.41% (4)	17.65% (3)	7.69% (7)
Vomiting/diarrhea/intestinal obstruction	5.41% (4)	0% (0)	4.40% (4)
Congenital infections (bacterial sepsis, congenital hepatitis, congenital herpes)	2.70% (2)	11.76% (2)	4.40% (4)
Hyperthyroidism, hypothyroidism, iodine deficiency	1.35% (1)	11.76% (2)	3.30% (3)
Neonatal cerebral depression	0% (0)	11.76% (2)	2.20% (2)
**Total**	**100% (74)**	**100% (17)**	**100% (91)**
**Neurodevelopmental disorders**			
Strabismus	50.91% (56)	32.26% (10)	46.81% (66)
Dyslexia, alexia, disorder of scholastic skills, disorders of speech and language	28.18% (31)	16.13% (5)	25.53% (36)
Disturbance of activity and attention with onset below 20 years	7.27% (8)	35.48% (11)	13.48% (19)
Mental retardation	7.27% (8)	6.45% (2)	7.09% (10)
Cerebral palsy	4.55% (5)	3.23% (1)	4.26% (6)
Autism	0.91% (1)	0% (0)	0.71% (1)
Down syndrome	0.91% (1)	0% (0)	0.71% (1)
Emotional disturbances, attachment disorder, and social anxiety	0% (0)	6.45% (2)	1.42% (2)
**Total**	**100% (110)**	**100% (31)**	**100% (141)**

**Table 2:** Characteristics of the merged cohort, the REGISTRY cohort, and the Enroll-HD cohort, divided by the group of participants, including sample sizes, gender ratio, the median age of diagnosis in years, median CAG length, and percentage and number of Caucasians, Europeans and North Americans. 95%CI, 95% confidence interval; HR, hazard ratio; IQR, interquartile range; N, the number of participants; %, the percentage of participants.

	Merged cohort	REGISTRY	Enroll-HD
**Reference group**			
N (N manifest)	13,631 (10,428)	9578 (7566)	4053 (2862)
% male	46.63%	46.82%	46.57%
Median CAG (IQR)	43 (41–45)	43 (41–45)	43 (41–45)
Median age of diagnosis (95%CI)	51.00 (50.68–51.32)	50.00 (49.63–50.37)	52.00 (51.43–52.57)
% Caucasian (N)	95.83% (13,062)	97.52% (9340)	90.20% (2656)
% Europe (N)	81.90% (11,164)	100% (9578)	39.13% (1586)
% North-America (N)	16.00% (2181)	0% (0)	53.81% (2181)
**Perinatal insults**			
N (N manifest HD)	91 (65)	74 (54)	17 (11)
% male	45.05%	47.30%	35.29%
Median CAG (IQR)	44 (42–47)	44 (42–47)	43 (41–44)
Median age of diagnosis (95%CI)	45.00 (42.07–47.93)	43.00 (39.84–46.16)	49.00 (43.57–54.43)
% Caucasian (N)	97.80% (89)	97.30% (72)	100% (17)
% Europe (N)	90.11% (82)	100% (74)	47.06% (8)
% North-America (N)	9.89% (9)	0% (0)	52.94% (9)
CAG-adjusted HR (95%CI)	1.61 (1.27–2.06)	1.64 (1.26–2.15)	1.45 (0.80–2.63)
**Neurodevelopmental disorders**			
N (N manifest HD)	141 (92)	110 (73)	31 (19)
% male	48.93%	52.72%	35.48%
Median CAG (IQR)	44 (42–46)	44 (42–46)	42 (40–44)
Median age of diagnosis (95%CI)	47.00 (43.63–50.37)	45.00 (41.36–48.64)	53.00 (47.30–58.70)
% Caucasian (N)	96.45% (136)	98.18% (108)	90.32% (28)
% Europe (N)	83.69% (118)	100% (110)	25.81% (8)
% North-America (N)	16.31% (23)	0% (0)	74.19% (23)
CAG-adjusted HR (95%CI)	1.41 (1.15–1.74)	1.61 (1.28–2.02)	0.24 (0.15–0.39)

Two-sided p-values below 0.05 were considered statistically significant. Statistical analyses were performed with IBM SPSS Statistics for Windows (Version 24.0, Armonk, NY).

## 3 Results

### 3.1 Description of comorbidities included

In the combined cohort, there were 91 participants with perinatal insults and 141 participants with neurodevelopmental disorders included. Seven cases had both a perinatal insult and a neurodevelopmental disorder. The comorbidities included in this study are listed in Table 1.

### 3.2 Influence of perinatal insults and neurodevelopmental disorders in disease-free survival

**Fig. 1:**
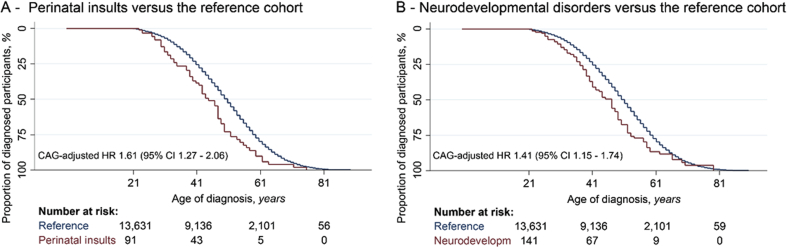
Kaplan-Meier survival curves of the age of diagnosis for the merged cohort. A Participants with perinatal insults versus the “reference” group; B Participants with neurodevelopmental disorders versus the “reference” group. 95% CI, 95% confidence interval; HR, hazard ratio; Neurodevelopm, neurodevelopmental disorders.

From the merged cohort, 13,856 GECs were included in our survival analysis, of which 10,578 had manifest HD and 3278 were pre-manifest carriers. The baseline characteristics of the reference groups were homogeneous for REGISTRY and Enroll-HD cohorts, and both were composed for the great majority of Caucasian participants (Table 2). The Kaplan Meier survival plot (Fig. 1) showed that both perinatal insults (PI) and neurodevelopmental disorders (ND) had a reduced time until an HD diagnosis (unadjusted HR's: 1.61 [95% CI: 1.26–2.05], 1.37 [95% CI: 1.11–1.68], respectively; CAG-adjusted HR's: 1.61 [95% CI: 1.27–2.06], 1.42 [95% CI: 1.16–1.75], respectively).

The overall time until diagnosis, characteristics and hazard ratios for each group are shown in Table 2. The secondary sensitivity analysis from the REGISTRY cohort confirmed these results for both the perinatal insults group and the neurodevelopmental disorders group (CAG-adjusted HR 1.64 [95% CI: 1.26–2.15], HR 1.63 [95% CI: 1.29–2.05], respectively). The Enroll-HD cohort showed non-statistically significant results (CAG-adjusted HR 1.45 [95% CI: 0.80–2.63], HR: 0.24 [95% CI: 0.15–0.39], respectively). We further investigated the relative contributions of specific disorders to the overall association seen. These analyses indicated that birth trauma and hypoxia, as well as meningitis or encephalitis, had the largest individual contributions to the overall association seen in perinatal insults group; however, the increased risk was still present after excluding these insults. In the neurodevelopmental disorders group, the contributions of individual insults to the overall association were considerably smaller. In this group ADHD and attention deficits had the largest individual effects on the overall association (Supplementary Table 1).

### 3.3 The association of gender and affected parent with disease-free survival per group

The overall time until diagnosis of HD by gender and affected parent is shown in Table 3. The interactions between group and gender, or group and affected parent were not statistically significant for either perinatal insults or neurodevelopmental disorders.

**Table 3:** Survival differences by gender and affected parent per group, including sample sizes, gender ratio, the median age of diagnosis in years, and median CAG length. 95%CI, 95% confidence interval; IQR, interquartile range; N, the number of participants.

	Reference	Perinatal insults	Neurodevelopmental disorders
**Effect of gender of participant on overall survival per group**			
**Males**			
N (N manifest HD)	6356 (5093)	41 (30)	69 (46)
Median age of diagnosis (95%CI)	50.00 (49.57–50.44)	43.00 (40.45–45.55)	48.00 (43.76–52.24)
Median CAG (IQR)	43 (4)	44 (4.5)	43 (5)
**Females**			
N (N manifest HD)	7275 (5335)	50 (35)	72 (46)
Median age of diagnosis (95%CI)	51.00 (50.54–51.55)	47.00 (44.26–49.75)	47.00 (43.09–50.91)
Median CAG (IQR)	43 (4)	43 (5.25)	44 (4)
**Comparison between genders**			
p-value	–	0.537	0.799
**Effect of gender of affected parent on overall survival per group**			
**Affected father**			
N (N manifest HD)	4747 (3476)	29 (22)	55 (34)
Median age of diagnosis (95%CI)	49.00 (48.46–49.54)	43.00 (41.50–44.50)	42.00 (34.67–49.33)
Median CAG (IQR)	43 (4)	44 (6)	44 (5)
**Affected mother**			
N (N manifest HD)	5359 (3675)	45 (27)	63 (37)
Median age of diagnosis (95%CI)	51.00 (50.47–51.53)	48.00 (41.02–54.98)	47.00 (44.11–49.89)
Median CAG (IQR)	43 (4)	44 (5)	43 (5)
**Comparison between affected parent**			
p-value (complete case analysis)	–	0.699	0.977
p-value (multiple imputation)	–	0.791	0.874

## 4 Discussion and conclusions

In this study, we explored the role of early-life events on the natural history of HD. Our results showed that perinatal insults and neurodevelopmental disorders were associated with earlier age of diagnosis of HD, with an observed unadjusted difference of 4–6 years. These differences are substantial since the most robust recently-described genetic modifiers, rs148491145 on chromosome 14 and rs146353869 on chromosome 15, accelerated the onset of disease by 3.2 and 6.1 years, respectively [5].

We further investigated the effect of gender or affected parent on the association, since the infant gender seems to modulate the risk of adverse outcomes after a perinatal insult in the general population and the risk of neurodevelopmental disorders [[16], [17], [18]]. The gender of the affected parent could also play a role through either the genetic anticipation phenomenon, where infants from affected father have a greater risk of CAG-repeat length expansion and henceforth an early symptomatic onset [14], or possibly the mother's genetic status may influence the pregnancy and associated *in utero* events. Our results showed that neither of these factors played a significant role in the overall association.

The accelerated diagnosis associated with perinatal and neurodevelopmental disorders could imply both biological and social factors. We speculated that early-life events may speed up the biological onset of HD through cumulative damage to the striatum and connected regions, which could diminish the neural reserve, accelerate neuropathology or alter neurodevelopment in a way that predisposes to earlier onset. The basal ganglia have a higher metabolic activity early in life, which makes this region especially vulnerable [19]. Early-life events also damage several other brain regions, including regions affected later in the course of HD, such as the cortico-thalamic circuitries [20], and cause lasting changes in epigenetic regulation of gene expression that may accelerate neurodegeneration decades later [21]. These biological factors could potentially aggravate the disease presentation and lead to earlier diagnosis, but need to be validated in other experimental models. Despite the clear temporal separation between perinatal and developmental problems and subsequent HD onset, our study design did not allow us to assess dose-response, specificity or experimental evidence from other biological systems, to confirm causality according to the Bradford Hill's criteria for causality in a biological system [22].

An alternate hypothesis is that early-life events affect social factors, such as more frequent interactions with medical care throughout life, which could lead to a diagnosis in an earlier stage of the disease. Infants that survive direct birth trauma, such as perinatal asphyxia often have a spectrum of neurological impairment, ranging from normal functioning to severe neurological disabilities; like cerebral palsy, attention deficit hyperactivity disorder (ADHD), autism, congenital hearing loss and neonatal seizures [13,15]. Preterm birth is commonly associated with cognitive, behavioral, attentional, or socialization deficits and occasionally with major motor deficits [23]. Preterm birth also increases the mortality risk in adulthood due to several health risks; including increased rates of diabetes, metabolic syndrome, neuropsychiatric disorders, respiratory, cardiovascular and kidney diseases [24]. All of these factors may increase the frequency of medical care, in support of the social theory.

In the general population, perinatal insults are often linked to neurodevelopmental disorders, such as learning difficulties, cognitive deficits or a developmental delay in approximately 60% of cases; cerebral palsy (21%); hearing impairment (20%); visual impairment (18%) or behavioral problems (11%) [13]. However, in HD, neurodevelopmental disorders only occurred in seven of the cases with perinatal insults. This may be due to incomplete reporting of neonatal insults in the neurodevelopmental group, or due to the multifactorial causes of neurodevelopmental disorders. Whilst perinatal insults increase the risk of strabismus, ADHD and dyslexia in the general population [17,25,26], these disorders are associated with several other environmental and genetic risk factors [25,27].

Surprisingly, we found a much lower incidence of perinatal insults in the HD groups than in the general population. The most common insults in the HD groups were direct birth trauma/asphyxia (with a prevalence of 0.20%), followed by preterm birth (0.12%), neonatal/congenital infections and kernicterus. In the general population asphyxia-related encephalopathy and preterm birth (before 37 weeks of gestation) respectively occurred in 0.85% [16] and 11.1% of live births globally in 2010 [28]. The most common neurodevelopmental disorders in the studied cohorts were strabismus (0.48%), disturbances in attention or activity (0.14%), and dyslexia or alexia (0.26%). The frequency of these disorders was also markedly lower than in the general population, where strabismus affects 2–3% [29], and ADHD and developmental dyslexia both affect around 7% of children below 18 years [18,30].

Our approach has limitations. The low frequency of perinatal and neurodevelopmental events in the studied cohorts may indicate that HD participants with additional comorbidities are less inclined to participate in research (selection bias), in addition to a possible recall bias due to difficulties in retrospectively assessing events which occurred several years before enrollment. Despite our use of a robust survival analysis, we could not eliminate the effects of these apparent biases, and the true effect of these insults may be smaller or larger than our study design could detect. The recall bias and relative rareness of these conditions meant that we had to combine perinatal insults and neurodevelopmental disorders that affect several divergent functional domains - such as language, attention, locomotion and cognition - into two main groups. These conditions are associated with dysfunction in several brain regions, and some conditions are hypothesized to be more detrimental to HD GECs than others. We assessed the relative contribution of some of the exposures to the overall association and conclude that birth trauma/hypoxia and meningitis/encephalitis had a larger contribution to the overall increase in risk than preterm birth or kernicterus. In the neurodevelopmental disorders ADHD had the largest effect, but the effect of individual insults on the overall hazard ratio was small. ADHD may mimic the earliest symptoms of HD, and thus we only included cases where ADHD was reported before 20 years. This was well before the average age of diagnosis of 47 years in the neurodevelopmental disorders group, and presumably the ADHD in these cases represented the neurodevelopmental disorder and not an early manifestation of HD. The association with neurodevelopmental disorders was not replicated in the Enroll-HD cohort, likely due to insufficient sample size and divergent participant characteristics. Despite these limitations, here we provide the first clinical association of early-life events with HD age of diagnosis.

In conclusion, this work shows that perinatal and neurodevelopmental insults associate with an earlier age at diagnosis of HD, with an effect comparable to that seen with known genetic modifiers. Further research is needed into the basis and mechanisms of this association to prove causation. These observations emphasize the far-reaching impact of early-life events in adult onset neurodegeneration.

## Author roles

Research project: A. Concept: MB, B. Design: MB, FR, C. Execution: MB, FR, AG. D. Data acquisition: EHDN Registry and Enroll-HD.

Statistical analysis: A. Design: BW, B. Analysis: MB, FR.

Manuscript: A. Drafting of the first version: MB. B. Review and critique: FBR, DA, BW, EW, BK, AG C. Supervision: EW, BK, AG.

## Financial disclosures

Melinda Barkhuizen: Reports no disclosures.

Filipe B Rodrigues: FBR has received honoraria from the International Parkinson's and Movement Disorders Society, and research funds from Fundaçao AstraZeneca and CHDI Foundation, Inc. His Host Institution, University College London Hospitals NHS Foundation Trust, has received funds as compensation for conducting clinical trials for Ionis Pharmaceuticals, Pfizer, and Teva Pharmaceuticals.

David Anderson: Reports no disclosures.

Bjorn Winkens: Reports no disclosures.

Edward J Wild: EJW has participated in scientific advisory boards with Hoffmann-La Roche Ltd, Ionis, Shire, GSK and Wave Life Sciences. All honoraria were paid through UCL Consultants Ltd, a wholly owned subsidiary of UCL. His Host Institution, University College London Hospitals NHS Foundation Trust, has received funds as compensation for conducting clinical trials for Ionis Pharmaceuticals, Pfizer, and Teva Pharmaceuticals.

Boris Kramer: Reports no disclosures.

Danilo Gavilanes: Reports no disclosures.

## Funding

The data for this study was freely supplied by the EHDN Registry and Enroll-HD initiatives, which are funded by the CHDI Foundation.

## Figures and Tables

**Multimedia component 4:**
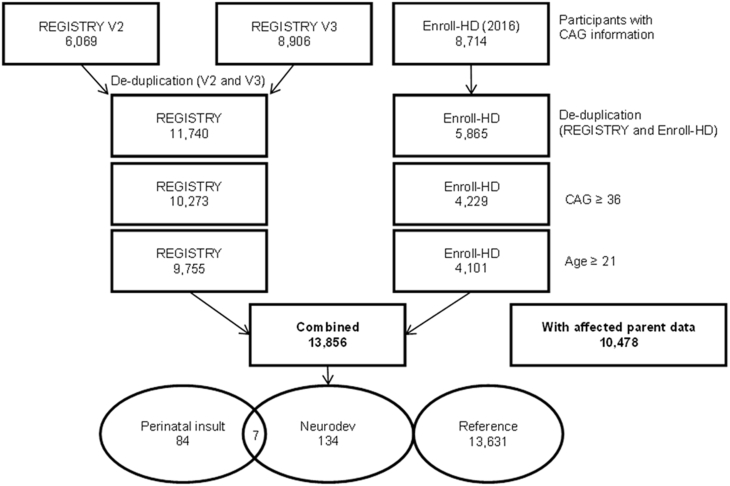
Flowchart of included participants and exclusions. Neurodev, neurodevelopmental disorders.
