# Supplementary material for: Perinatal insults and neurodevelopmental disorders may impact Huntington's disease age of diagnosis
Source: Parkinsonism Relat Disord. 2018 Oct;55:55–60. doi: 10.1016/j.parkreldis.2018.05.016 (PMC6226577; doi:10.1016/j.parkreldis.2018.05.016)
Supplement: Multimedia component 2. [file mmc2.pdf]

Enroll-HD

An Acknowledgement list with email addresses is available to qualified researchers
CLICK HERE to become a qualified researcher

Site	Country	Name
AarhusUnivHosp	Denmark	Anette Torvin Møller
AarhusUnivHosp	Denmark	Louise Hasselstrøm Madsen
AucklandCityHosp	New Zealand	Richard Roxburgh
AucklandCityHosp	New Zealand	Virginia Hogg
AucklandCityHosp	New Zealand	Richard Roxburgh
AucklandCityHosp	New Zealand	Virginia Hogg
AugustaUniv	USA	John Morgan
AugustaUniv	USA	Paula Jackson
AvonWiltMenHeaPartTr	UK	Lesley Gowers
AvonWiltMenHeaPartTr	UK	Carol Hall
AyrshireHealthBoard	UK	Sharon Mulhern
AyrshireHealthBoard	UK	Margo Henry
AyrshireHealthBoard	UK	Tim Johnston
AziendaOspedSanAndre	Italy	Michela Ferraldeschi.
AziendaOspedSanAndre	Italy	Giovanni Ristori
AziendaOspedSanAndre	Italy	Silvia Romano
BaylorCollMed	USA	Ami Patel
BaylorCollMed	USA	Christine Hunter
BaylorCollMed	USA	Joseph Jankovic, MD
BeaumontHosp	Ireland	Ms Fiona O'Donovan
BeaumontHosp	Ireland	Prof Orla Hardiman
BeaumontHosp	Ireland	Dr Sinead Maguire
BeaumontHosp	Ireland	Dr Samira Bouazzaoui
BeaumontHosp	Ireland	Niall Pender
BirmSolNHSFounTrust	UK	Ellice Parkinson
BirmSolNHSFounTrust	UK	Hugh Rickards
BostonMedCtr	USA	Raymond James
BostonMedCtr	USA	Marie Saint-Hilaire
BurgosFoun	Spain	Esther Cubo
BurgosFoun	Spain	Natividad Mariscal
CardiffUniv	UK	Anne Rosser
CardiffUniv	UK	Rebecca Cousins
CardiffUniv	UK	Thomas Massey
CardiffUniv	UK	Duncan McLauchlan
CardiffUniv	UK	Monica Busse
CenterMovDis	Canada	Jonielyn Carlos
CenterMovDis	Canada	Kimberly Thompson
CenterMovDis	Canada	Mark Guttman
CentHospUnivMontreal	Canada	Lyne Jean

CentHospUnivMontreal	Canada	Sylvain Chouinard
CentManHospFounTrust	UK	Zara Skitt
CentManHospFounTrust	UK	Siofra Peeren
CentManHospFounTrust	UK	David Craufurd
CentManHospFounTrust	UK	Dawn Rogers
CentManHospFounTrust	UK	Iris Trender-Gerhard
CentManHospFounTrust	UK	Liz Howard
CETRAM	Chile	Maria Consuelo Moos
CETRAM	Chile	Pedro Chana
ClevelandClinicFoun	USA	Anwar Ahmed
ClinTriCtrMaastricht	Netherlands	Mayke Oosterloo
ClinTriCtrMaastricht	Netherlands	Mirella Davies-Waber
ColumbiaUniv	USA	Ronda Clouse
ColumbiaUniv	USA	Massood Manoochehri
ColumbiaUniv	USA	Sarah Janicki
ColumbiaUniv	USA	Pietro Mazzoni
ColumbiaUniv	USA	Elan Louis
ColumbiaUniv	USA	Karen Marder
ColumbiaUniv	USA	Paula Wasserman
CooperHealth	USA	Amy Colcher
CooperHealth	USA	Andrew March
CopernicusPodLec	Poland	Agnieszka Konkel
CopernicusPodLec	Poland	Witold Soltan
CrucesHosp	Spain	Koldo Berganzo Corrales
CrucesHosp	Spain	Maria Angeles Acera Gil
DukeUniv	USA	Peggy Perry-Trice
DukeUniv	USA	Burton Scott
EmoryUniv	USA	Elaine Sperin
EmoryUniv	USA	Jaime Hatcher-Martin
EmoryUniv	USA	Stewart Factor
FifeHealthBoard	UK	Gareth Thomas
FifeHealthBoard	UK	Nicola Johns
GeorgeHuntingtonInst	Germany	Herwig Lange
GeorgeHuntingtonInst	Germany	Laura Dornhege
GeorgeHuntingtonInst	Germany	Paula Raulet
GeorgeHuntingtonInst	Germany	Ralf Reilmann
GeorgeHuntingtonInst	Germany	Stefan Bohlen
GeorgetownUniv	USA	Karen Anderson
GeorgetownUniv	USA	Natasha Scott
GreatGlasgowHealthBoard	UK	Catherine Deith
GreatGlasgowHealthBoard	UK	Dr. Stuart Ritchie
GuyandStThomFounTrust	UK	Deborah Ruddy
GuyandStThomFounTrust	UK	Dene Robertson
GuyandStThomFounTrust	UK	Alison Lashwood
GuyandStThomFounTrust	UK	Elizabeth White
GuyandStThomFounTrust	UK	Thomasin Andrews
HNDC	USA	Gregory Suter

HNDC	USA	William M Mallonee
HospCreuSantPau	Spain	Andrea Horta
HospCreuSantPau	Spain	Jaime Kulisevsky
HospInfantChrisBadaj	Spain	Carmen Durán Herrera
HospInfantChrisBadaj	Spain	Patrocinio García Moreno
HospMareMerce	Spain	Elvira Roca Goma
HospMareMerce	Spain	Jesús Miguel Ruíz Idiago
HospUnivBellvitge	Spain	Matilde Calopa
HospUnivBellvitge	Spain	Jordi Bas
InstNeuroBuenoAires	Argentina	Emilia Gatto
InstPsychandNeuro	Poland	Grzegorz Witkowski
InstPsychandNeuro	Poland	Iwona Stepniak
JimenDiazFoun	Spain	Pedro J Garcia Ruiz
JimenDiazFoun	Spain	Asunción Martinez
JohnsHopkinsUniv	USA	Frederick C. Nucifora Jr.
JohnsHopkinsUniv	USA	Christopher Ross
JohnsHopkinsUniv	USA	Mollie Jenckes
KboIsarAmpKlinTauf	Germany	Alzbeta Mühlbäck
KboIsarAmpKlinTauf	Germany	Matthias Dose
KboIsarAmpKlinTauf	Germany	Michael Bachmaier
KboIsarAmpKlinTauf	Germany	Ralf Marquard
KrakowskaAkademiaNeuro	Poland	Monica Rudzinska
KrakowskaAkademiaNeuro	Poland	Natalia Grabska
LeedsTeachHospTrust	UK	Alison Kraus
LeedsTeachHospTrust	UK	Stuart Jamieson
LeedsTeachHospTrust	UK	Ivana Markova
LeedsTeachHospTrust	UK	Emma Hobson
LeedsTeachHospTrust	UK	Callum Schofield
LegaItalRiceHunt	Italy	Massimo Marano
LegaItalRiceHunt	Italy	Simone Migliore
LegaItalRiceHunt	Italy	Sabrina Maffi
LegaItalRiceHunt	Italy	Barbara D'Alessio
LegaItalRiceHunt	Italy	Ferdinando Squitieri
Leicestershire	UK	Dawn Freire-Patino
Leicestershire	UK	Caroline Hallam
Leicestershire	UK	Reza Kiani
LeidenUniv	Netherlands	Raymund Roos
LeidenUniv	Netherlands	Marye Hogenboom
LomaLindaUniv	USA	Dharmaseeli Moses
LothianHealthBoard	UK	Philip Greene
LothianHealthBoard	UK	Marie McGill
LothianHealthBoard	UK	Mary Porteous
MilanGenetic	Italy	Anna Castaldo
MilanGenetic	Italy	Caterina Mariotti
MilanGenetic	Italy	Lorenzo Nanetti
MilanNeuro	Italy	Dominga Paridi
MilanNeuro	Italy	Paola Soliveri

MilanNeuro	Italy	Simona Castagliuolo
MinnMedResFoun	USA	Dawn Radtke
MinnMedResFoun	USA	Martha Nance
MonashUniv	Australia	Dr. Andrew Churchyard
MonashUniv	Australia	Katie Fitzgerald
MonashUniv	Australia	Julie Stout
NHSForthValley	UK	Christian Neumann
NHSForthValley	UK	David Thomson
NorStaffCombHeaTrust	UK	George El-Nimr
NorStaffCombHeaTrust	UK	Karen Kennedy
NorthBristolTrust	UK	Dr Catherine Pennington
NorthBristolTrust	UK	Serena Dillon
NorthBristolTrust	UK	Elizabeth Coulthard
NorthBristolTrust	UK	Louise Gethin
NorthMetroHlthServ	Australia	Jacenta Abbott
NorthMetroHlthServ	Australia	Peter Panegyres
NorthumbTyneFreeman	UK	Jill Davison
NorthumbTyneFreeman	UK	Suresh Komati
NorthumbTyneFreeman	UK	Sarah Edwards
OhioStateUniv	USA	Allison Daley
OhioStateUniv	USA	Sandra Kostyk
OhioStateUniv	USA	Katherine Ambrogi
OxfordUnivHospTrust	UK	Professor Andrea H Nemeth
OxfordUnivHospTrust	UK	Sarsha Wilson
PlyHospNHSTrust	UK	Julie Frost
PlyHospNHSTrust	UK	Dr. Rupert Noad
PlyHospNHSTrust	UK	Leanne Timings
PooleHospFounTrust	UK	Annemieke Fox
PooleHospFounTrust	UK	John Burn
PoznanUniv	Poland	Daniel Zielonka
PoznanUniv	Poland	Elżbieta Alicja Puch
RamonCajalUnivHosp	Spain	José Luis López-Sendón Moreno
RamonCajalUnivHosp	Spain	Verónica Mañanes Barral
RockyMtnMovDis	USA	Jessica Jaynes
RockyMtnMovDis	USA	Rajeev Kumar
RoyalDevExetFounTrst	UK	Sarah Irvine
RoyalDevExetFounTrst	UK	Timothy Harrower
RoyBerkNHSFounTrust	UK	Anita Foster
RoyBerkNHSFounTrust	UK	Dr. Richard Armstrong
RushUniv	USA	Courtney Timms
RushUniv	USA	Jennifer Goldman
RutgersUniv	USA	Daniel Schneider
RutgersUniv	USA	Deborah Caputo
SanfordResearch	USA	Tish Skarloken
SanfordResearch	USA	Tanya Harlow
SanfordResearch	USA	Destini Spaeth
SchleswigHolsteinHosp	Germany	Sandra Bloess

SchleswigHolsteinHosp	Germany	Alexander Münchau
SchleswigHolsteinHosp	Germany	Jenny Schmalfeld
SchleswigHolsteinHosp	Germany	Klaus Gehring
SchleswigHolsteinHosp	Germany	Vera Tadic
SheffieldChildFouTru	UK	Anya Kholkina
SheffieldChildFouTru	UK	Oliver Quarrell
SilesianMedUnivKatowice	Poland	Klaudia Plinta
SonEspasesHosp	Spain	Penélope Navas Arques
SonEspasesHosp	Spain	Ines Legarda
SouthamptonUnivHospTrust	UK	Christopher Kipps
SouthamptonUnivHospTrust	UK	Veena Agarwal
StAndrewsHealth	UK	Elvina Chu
StGeorgeHealthTrust	UK	Nayana Lahiri
StGeorgeHealthTrust	UK	Uruj Anjum
StJosefAndElisabethHosp	Germany	Barbara Kaminski
StJosefAndElisabethHosp	Germany	Carsten Saft
StJosefAndElisabethHosp	Germany	Rainer Hoffmann
StJosefAndElisabethHosp	Germany	Sarah von Hein
Tayside	UK	Alison Tonner
Tayside	UK	Lindsay Wilson
Tayside	UK	David Goudie
Tayside	UK	Paula McFadyen
TechUnivMunich	Germany	Adolf Weindl
TechUnivMunich	Germany	Antje Lüsebrink
UnivAberdeen	UK	Daniela Rae
UnivAberdeen	UK	Alisdair Ross
UnivAberdeen	UK	Stella Sihlabela
UnivAberdeen	UK	Zosia Miedzybrodzka
UnivAlbertaGlenrose	Canada	Pam King
UnivAlbertaGlenrose	Canada	Wayne Martin
UnivBari	Italy	Marina de Tommaso
UnivBari	Italy	Vittorio Sciruicchio
UnivBologna	Italy	Cesa Scaglione
UnivBologna	Italy	Pietro Cortelli
UnivBritishCol	Canada	Allison Coleman
UnivBritishCol	Canada	Lynn Raymond
UnivBritishCol	Canada	Blair Leavitt
UnivCalDavis	USA	Alexandra (Sasha) Duffy
UnivCalDavis	USA	Amanda Martin
UnivCalDavis	USA	Ashok Joshua Dayananthan
UnivCalDavis	USA	Vicki Wheelock
UnivCalgary	Canada	Lorelei Tainsh (Derwent)
UnivCalgary	Canada	Sarah Furtado
UnivCalIrvine	USA	Nicolas Phielipp
UnivCalIrvine	USA	Durk Thompson
UnivCalIrvine	USA	Breana Chew
UnivCalSanDiego	USA	Jody Corey-Bloom

UnivCalSanDiego	USA	Sungmee Park
UnivCalSanDiego	USA	Ajay Nathan
UnivCalSanFran	USA	Alexandra Nelson
UnivCambridge	UK	Dr Sarah Mason
UnivCambridge	UK	Dr Caroline Williams-Gray
UnivCambridge	UK	Anna Gerritz (nee Di Pietro)
UnivCambridge	UK	Roger Barker
UnivCattolicaSacrCur	Italy	Flavia Torlizzi
UnivCattolicaSacrCur	Italy	Anna Rita Bentivoglio
UnivCattolicaSacrCur	Italy	Marcella Solito
UnivCharite	Germany	Josef Priller
UnivCharite	Germany	Anika Langenfurth
UnivCharite	Germany	Markus Beuth
UnivChicago	USA	Joan Young
UnivChicago	USA	Tao Xie
UnivCincinnatiPhysCo	USA	Andrew Duker
UnivCincinnatiPhysCo	USA	Katie Krier
UnivCollLondon	UK	Ed Wild
UnivCollLondon	UK	Monica Lewis
UnivCollLondon	UK	Nicola Robertson
UnivCollLondon	UK	Sarah Tabrizi
UnivConnHealthCtr	USA	Bonnie Hennig
UnivConnHealthCtr	USA	Kevin James Manning
UniverMedCtrFreiburg	Germany	Gerit Kammel
UniverMedCtrFreiburg	Germany	Stephan Klebe
UniverMedCtrFreiburg	Germany	Michel Rijntjes
UnivGroningen	Netherlands	H.P.H. Kremer
UnivGroningen	Netherlands	Jesper Klooster
UnivHospAachen	Germany	Beate Schumann
UnivHospAachen	Germany	Johannes Schiefer
UnivHospAachen	Germany	Kathrin Reetz
UnivHospCopenhagen	Denmark	Christina Vangsted Hansen
UnivHospCopenhagen	Denmark	Jørgen Nielsen
UnivHospCopenhagen	Denmark	Lena E. Hjermind
UnivHospCopenhagen	Denmark	Suzanne Granhøj Lindquist
UnivHospCopenhagen	Denmark	Peter Roos
UnivHospErlangen	Germany	Susanne Seifert
UnivHospErlangen	Germany	Christina Kozay
UnivHospErlangen	Germany	Zacharias Kohl
UnivHospGiessenMarburg	Germany	Katrin Bürk
UnivHospOdense	Denmark	Lene Wermuth
UnivHospOdense	Denmark	Marianne Dybro Lundsgaard
UnivHospUlm	Germany	Hela Jerbi
UnivHospUlm	Germany	Jan Lewerenz
UnivHospUlm	Germany	Michael Orth
UnivHospUlm	Germany	Panteha Fathinia
UnivHospUlm	Germany	Patrick Weydt

UnivHospWuerzburg	Germany	Kerstin Nöth
UnivHospWuerzburg	Germany	Christine Leypold
UnivHospWuerzburg	Germany	Kai Boelmans
UnivIllinois	USA	Mitch King
UnivIllinois	USA	Sadie Foster
UnivIowa	USA	Angel L. Dominguez
UnivIowa	USA	Jane S Paulsen
UnivKansasMedCtrResInst	USA	Carolyn Gray
UnivKansasMedCtrResInst	USA	Richard Dubinsky
UnivMaryland	USA	Terra Hill
UnivMaryland	USA	William Keller
UnivMich	USA	Elizabeth Sullivan
UnivNaples	Italy	Luigi di Maio
UnivNaples	Italy	Cinzia Valeria Russo
UnivNaples	Italy	Silvio Peluso
UnivNaples	Italy	Elena Salvatore
UnivNaples	Italy	Giuseppe De Michele
UnivOtago	New Zealand	Laura Paermentier
UnivOtago	New Zealand	Tim Anderson
UnivOtago	New Zealand	Laura Paermentier
UnivOtago	New Zealand	Tim Anderson
UnivPitt	USA	Larry Ivanco
UnivPitt	USA	Valerie Suski
UnivRochester	USA	Amy Chesire
UnivRochester	USA	Frederick Marshall
UnivRochester	USA	Julia Iourinets
UnivSouthFlorida	USA	Danielle Hergert
UnivSouthFlorida	USA	Patricia Johnson
UnivSouthFlorida	USA	Emily Kellogg
UnivSouthFlorida	USA	Juan Sanchez-Ramos
UnivSouthFlorida	USA	Kelly (Kollen) Elliott
UnivTenn	USA	Dr. Mark LeDoux
UnivTenn	USA	Amanda Nolte
UnivTexasHlthCntrHous	USA	Erin Furr Stimming
UnivTexasHlthCntrHous	USA	Leigh Beth Latham
UnivUtah	USA	Meghan Zorn
UnivUtah	USA	Matthew Halverson
UnivUtah	USA	Stefan Pulst
UnivUtah	USA	Paola Wall
UnivVermont	USA	Emily Houston
UnivVermont	USA	James Boyd
UnivVirginia	USA	Katie L. Sullivan
UnivVirginia	USA	Susan Dietrich
UnivWarsaw	Poland	Piotr Janik
UnivWarsaw	Poland	Zygmunt Jamrozik
UnivWash	USA	Ali Samii
UnivWash	USA	Debra Del Castillo

VanderbiltUniv	USA	Daniel O. Claassen
VanderbiltUniv	USA	Lauren West
VanderbiltUniv	USA	Onyebuchi Okeke
VirginiaCommUniv	USA	Claudia Testa
VirginiaCommUniv	USA	Ginger Norris
WakeForestUniv	USA	Christine O'Neill
WakeForestUniv	USA	Francis Walker
WaltonCtrFounTrust	UK	Louise Pate
WaltonCtrFounTrust	UK	Rhys Davies
WashingtonUniv	USA	Joel S. Perlmutter
WashingtonUniv	USA	Stacey Barton
WashingtonUniv	USA	Elaine Most
WestSydneyHlthDist	Australia	Dr. Clement Loy
WestSydneyHlthDist	Australia	Jillian McMillan
WestSydneyHlthDist	Australia	Therese Alting
