# Supplementary material for: Perinatal insults and neurodevelopmental disorders may impact Huntington's disease age of diagnosis
Source: Parkinsonism Relat Disord. 2018 Oct;55:55–60. doi: 10.1016/j.parkreldis.2018.05.016 (PMC6226577; doi:10.1016/j.parkreldis.2018.05.016)
Supplement: Multimedia component 3. [file mmc3.docx]

|  | CAG-adjusted HR (95%CI) | Median age of diagnosis (95%CI) |
| --- | --- | --- |
| Perinatal insults | | |
| Overall | 1.61 (1.27-2.06) | 45.00 (42.07-47.93) |
| Excluding birth injury, birth asphyxia, apnea or meconium aspiration | 1.53 (1.15-2.03) | 45.00 (40.31-49.69) |
| Excluding preterm birth | 1.66 (1.26-2.18) | 47.00 (43.70-50.30) |
| Excluding kernicterus | 1.64 (1.26- 2.13) | 44.00 (39.43-48.57) |
| Excluding meningitis and encephalitis | 1.50 (1.16-1.95) | 47.00 (43.82-50.18) |
| Neurodevelopmental disorders | | |
| Overall | 1.42 (1.16-1.75) | 47.00 (43.38-50.62) |
| Excluding ADHD and attention disorders | 1.38 (1.11-1.72) | 47.00 (43.45-50.55) |
| Excluding dyslexia and learning disorders | 1.45 (1.11-1.84) | 44.00 (40.14-47.86) |
| Excluding strabismus | 1.45 (1.10-1.91) | 47.00 (42.80-51.20) |
| Excluding mental retardation | 1.43 (1.16-1.77) | 47.00 (43.27-50.73) |
| Excluding cerebral palsy | 1.42 (1.16-1.75) | 47.00(43.38-50.62) |
